# Fully coupled patient-specific fluid–structure interaction modeling of post-TAVI hemodynamics compared with echocardiographic measurements

**DOI:** 10.1007/s10237-026-02130-1

**Published:** 2026-09-04

**Authors:** Chiara Catalano, Deniz Ozturk, Maria Bastron, Alessandra Zerillo, Stefano Cannata, Caterina Gandolfo, Salvatore Pasta

**Affiliations:** 1 Department of Research, IRCCS ISMETT, Via Tricomi, 5, Palermo, Italy; 2 Medividia, Herent, Belgium; 3 Department for the Treatment and Study of Cardiothoracic Diseases and Cardiothoracic Transplantation, IRCCS ISMETT, Via Tricomi, 5, Palermo, Italy; 4 Department of Engineering, Università Degli Studi Di Palermo, Viale Delle Scienze Ed. 8, Palermo, Italy

**Keywords:** Transcatheter aortic valve implantation, Fluid–structure interaction, Patient-specific modeling, Cardiovascular biomechanics

## Abstract

To develop a fully coupled, patient-specific fluid–structure interaction (FSI) framework for quantitative assessment of post–transcatheter aortic valve implantation (TAVI) hemodynamics and valve biomechanics, and to compare selected simulation-derived hemodynamic indices with post-procedural echocardiographic measurements. Patient-specific geometries were reconstructed from pre-operative computed tomography angiography in five subjects treated with SAPIEN 3 Ultra (S3) devices. Structural TAVI deployment was simulated using Abaqus/Explicit and subsequently coupled with FlowVision for performing a two-way post-TAVI FSI analysis. Personalized boundary conditions were derived from clinical measurements, including heart rate, blood pressure, and echocardiographic flow data. Predicted peak velocity, effective orifice area (EOA), and transvalvular pressure gradients (TPG) were quantitatively compared with post-procedural echocardiography using empirical cumulative distribution functions and area-based error metrics. The FSI framework reproduced realistic leaflet kinematics and patient-specific flow patterns, highlighting marked inter-patient variability despite identical device types. Average of predicted peak systolic velocity (2.66 ± 0.59 m/s) and TPG (22 ± 10.7 mmHg) showed good agreement with echocardiographic measurements as the area metric was below 10% for both peak TPG and velocity. Larger discrepancies were observed for EOA due to patient variability and reliability of echocardiographic measurements. The model additionally quantified biomechanical and hemodynamic parameters, including leaflet stress, time-averaged wall shear stress (TAWSS), and blood residence time (BRT). The proposed fully coupled FSI framework enables patient-specific analysis of post-TAVI hemodynamics and valve mechanics for S3 balloon-expandable intra-annular system. The preliminary comparison with echocardiographic measurements showed good agreement for peak velocity and peak TPG, while larger EOA discrepancies highlight the need for further clinical benchmarking using larger cohorts and more direct measurement modalities.

## Introduction

Transcatheter aortic valve implantation (TAVI) has become an established therapy for patients with symptomatic aortic stenosis across a broad spectrum of surgical risk (Lim 2023; Braghiroli et al. 2020). As device designs evolve and procedural indications expand in young individuals, increasing attention is being devoted to post-implantation transvalvular hemodynamics performance. Residual transvalvular pressure gradients, abnormal flow patterns, and elevated shear stresses have been associated with potential endothelial damage and valve degeneration (Dasi et al. 2009; Salmasi et al. 2021). However, detailed characterization of these phenomena remains limited by imaging resolution, measurement uncertainty, and the inability to directly observe leaflet–flow interaction under in vivo conditions.

Advances in numerical modeling, fluid–structure interaction (FSI) analyses overcome these limitations providing accurate and physiologically realistic assessments of post-TAVI hemodynamics. Many studies (Kandail et al. 2018; Ghosh et al. 2020a; Pasta et al. 2021; Oks et al. 2024; Pan et al. 2024) leveraged FSI combining structural and fluid dynamical models to better simulate the intervention. Kandail and collaborators (2018) characterized self-expanded hemodynamics simulating annular and supra-annular deployment with personalized geometries and realistic boundary conditions. Oks et al. (2024) proposed patient-specific FSI models simulating TAVI with varying sinotubular junction diameters to evaluate their impact on post-deployment hemodynamics and thrombogenicity. Ghosh and colleagues (2020a) quantified post-TAVI paravalvular leakage for different implantation depths via FSI simulations. However, most of these studies did not perform direct reconstruction of native aortic valve leaflets from pre-operative scans, neglected the inclusion of calcifications and native leaflets, or did not account for personalized boundary conditions. Despite the growing number of FSI-based investigations on TAVI, quantitative comparison of patient-specific simulation outputs with routinely acquired post-procedural clinical measurements remains limited. Specifically, many studies focus on reproducing post-operative flow patterns, whereas only a subset report direct comparisons with patient-specific hemodynamic indices. A comparative summary table of key FSI-based studies on transcatheter heart valves, including valve type, numerical methodologies, and key findings, is presented (see Table 1).

**Table 1:** Studies using fully coupled FSI for transcatheter heart valves focused on post-TAVI hemodynamics

Author	Numerical method	Device	Structural output	Hemodynamic output	Key findings
Wu et al. (2019)	Immersogeometric Analysis	CoreValve, 26 mm	Radial outward force, static friction force, strain	WSS, velocity fields, flow rate, pressure	Friction-to-radial force ratio (~ 0.22) indicates migration risk
Brown et al. (2023)	Immersed FE-Difference (IFED)	Evolut R, 26 mm	Mises stress, leaflet tip displacement	TPG, flow rate, EOA, velocity	Captured paravalvular leaks; simulated EOA and gradients matched clinical data
Fumagalli et al. (2023)	Arbitrary Lagrangian–Eulerian	SAPIEN XT, 23 mm	Mises stress in the annulus, structural displacement	TAWSS critical areas, velocity field, pressure drop	High TAWSS critical areas correlate with structural valve deterioration
Luraghi et al. (2019)	Operator split Arbitrary Lagrangian–Eulerian	Evolut R, 29 mm	Principal stress, contact pressure	Velocity field, orifice area, flow rate	Accurately predicted leakage severity; validated with Doppler traces and post-op imaging
Ghosh et al. (2020b)	Partitioned FSI (Abaqus + FlowVision)	Evolut R, 26 mm	Anchoring contact area and contact force	leakage volume, EOA, stroke volume, oscillatory shear index	Implantation at a ventricular position provided optimal performance by reducing thrombogenic potential and leakage
Kandail et al. (2018)	SGGR-based FSI	CoreValve, 29 mm	Prosthesis valve configuration	Velocity magnitude, WSS, coronary flow rate	Supra-annular deployment tilted the device, shifting high WSS regions downstream into the ascending aorta

HR indicates heart rate; Psys and Pdias indicate systolic and diastolic brachial blood pressure, respectively; velocity indicates peak post-TAVI transvalvular velocity measured by echocardiography; peak TPG and mean TPG indicate peak and mean transvalvular pressure gradients measured by echocardiography

In this study, we developed a fully coupled patient-specific FSI framework for post-TAVI hemodynamic analysis in patients treated with 23 mm and 26 mm SAPIEN 3 Ultra (S3) transcatheter heart valves (Edwards Lifesciences, Irvine, CA, USA). The approach integrates personalized anatomical reconstruction, clinically calibrated boundary conditions, and quantitative comparison with post-procedural echocardiographic measurements. Simulation-derived peak velocity, EOA, and TPG were compared with clinical measurements using cumulative distribution functions and area-based difference metrics. This comparison was not intended to constitute full clinical validation of the complete modeling pipeline, but rather to assess the consistency of selected hemodynamic outputs with routinely available clinical data.

## Methods

The patient-specific TAVI model was developed according to the verification, validation, and uncertainty quantification (VVUQ) principles reported by the ASME V&V40. Details on the validation of the patient-specific segmentation process and details on the structural model accuracy using the VVUQ are reported in our previous studies (Catalano et al. 2025; Scuoppo et al. 2025). Here, we focus on the fully-coupled post-TAVI simulation and its validation against clinical data in five patients.

### Patient study group

Five patients underwent TAVI with both the 23 mm and 26 mm Sapien 3 (S3) Ultra device were investigated. Here, the abbreviation S3 refers specifically to the S3 Ultra balloon-expandable intra-annular transcatheter heart valve and not to other SAPIEN platforms. After obtaining ethical approval and informed consent, images from computed tomography angiography (CTA) were collected prior to TAVI procedures for segmentation. Pre-procedural CTA was performed using an ECG-gated contrast-enhanced acquisition protocol on a 256-slice dual-source CT scanner (SOMATOM Force, Siemens Healthineers, Forchheim, Germany). Images were reconstructed with a slice thickness of 0.475 mm and an image matrix of 512 × 512 pixels. Iodinated contrast agent (approximately 1.8 mL per kg) was administered intravenously according to the institutional TAVI planning protocol. A dedicated calcium-scoring CT acquisition was also performed as part of the clinical assessment but was not used for computational model development. Calcific plaques were segmented from the contrast-enhanced CT angiography images. After TAVI procedure, transesophageal echocardiography was carried out as part of clinical care to quantify flow velocity across S3, effective orifice area (EOA), and transvalvular pressure gradient (TPG) at peak and over the cardiac cycle. Additionally, heart rate and brachial blood pressure prior to TAVI procedure were collected for tuning patient-specific flow boundary conditions. All patients presented with aortic stenosis, graded from moderate to severe according to pre-procedural clinical assessment. One patient had combined aortic stenosis and aortic regurgitation. Table 2 summarizes clinical data and post-procedural echocardiographic assessment of TAVI procedures.

**Table 2:** Patient demographic data alongside with device size and post-TAVI echocardiographic measurements

	S3 size (mm)	HR (bpm)	P_sys_ (mmHg)	P_dias_ (mmHg)	Velocity (m/s)	Peak TPG (mmHg)	Mean TPG (mmHg)
Case A	23	88	118	58	2.7	30	16
Case B	26	70	105	55	2.1	18	7
Case C	23	70	147	37	2.6	28	14
Case D	23	65	100	50	2.7	30	15
Case E	26	61	137	66	3.3	45	21

### Patient-specific TAVI models

Patient-specific anatomical models were segmented from end-diastolic CTA images as described previously by our group (Catalano et al. 2024). In brief, semi-automatic thresholding followed my manual editing and smoothing of the luminal wall was adopted for the root model. A single fixed HU threshold was not used for all patients. Segmentation was adapted to each patient according to the gray-intensity distribution of the contrast-enhanced CT images, as described in our previous segmentation study (Scuoppo et al. 2024). Briefly, the aortic lumen was segmented using a seed-based thresholding approach, with patient-specific seed values ranging from 326 to 1287 HU and automatic mask propagation stopped when neighboring voxels differed from the seed value by more than ± 78 HU. Calcific plaques were segmented using automatic thresholding with intensity values in the range of 850–2145 HU. As the aortic valve is not detectable on the CTA scan, native leaflets at diastolic phase were modeled using anatomic landmarks and 3rd-order NURBS curves in Rhinoceros software (Rhinoceros v.7, McNeel & associates, USA) (Scuoppo et al. 2024). A specific study was carried out to quantify the accuracy of the proposed segmentation process using the ISO:50 thresholding method as the reference segmentation process and pre-TAVI end-systolic medical images as used for the evaluation of segmentation accuracy (Scuoppo et al. 2024). It should be also noted that all segmentations were performed by the same experienced operator.

Numerical verification of mesh topology and solver setting was carried out to improve model quality. Specifically, the aortic root was discretized using triangular shell elements of 0.6 mm with reduced integration (S3R). Calcifications were meshed with unstructured tetrahedral solid elements with size of 0.5 mm (C3D4). Native valve leaflets were assigned a uniform thickness of 0.3 mm and discretized using three layers of prismatic elements (C3D6) through the thickness, resulting in an approximate through-thickness element size of 0.1 mm. The in-plane characteristic element size was 0.3 mm. For material modeling, the Neo-Hookean constitutive formulation was adopted for both the aortic root and native valve leaflets, with the material descriptor properly tuned for each patient using a regression-based approach and the systolic CTA cardiac phase (Catalano et al. 2024). Calcific plaques were modeled using a nearly incompressible Neo-Hookean formulation. The Neo-Hookean constants were derived from linear elastic properties reported from Bosi et al. (2018) and thus by converting Young’s modulus and Poisson’s ratio into the equivalent Abaqus Neo-Hookean parameters (C10 = 67.7 MPa and D1 = 7.5E−3 MPa-1). This formulation was adopted to improve numerical stability during simulations. The resulting patient-specific TAVI model was validated and its uncertainty quantified, showing predictive capabilities on the structural device deployment with errors < 10% against post-TAVI CTA images as reported previously (Catalano et al. 2025; Scuoppo et al. 2025).

### Structural TAVI simulation

The S3 stent frame model was modeled with solid brick element (C3D8R) with geometry acquired by micro-CT imaging as described in a previous study (Pasta et al. 2020). The cobalt–chromium material behavior of the stent frame was modeled using isotropic elasticity (E = 238.54 GPa, *ν* = 0.29) and Johnson–Cook plasticity (A = 465 MPa, B = 2140 MPa, *n* = 0.73, *ρ*= 7,650 kg/m^3^) as in our previous study (Catalano et al. 2024). Device valve leaflets were modeled with solid elements (size of 0.3 mm, C3D8R) and a second-order Ogden constitutive law (m1 = 0.96 MPa, a1 = − 56.5, m2 = 3.57 MPa, a2 = 1.87, and D = 0.027 MPa-1) as reported by Catalano et al. (2024). Differently, the inner and outer skirts of S3 model had a Neo-Hookean material behavior (C1 = 1.7 MPa and D = 0.65 MPa-1) and thickness of 0.2 mm. Tie constraints were used to model sutures connecting the stent frame with both the skirt and valve leaflets. These constraints were applied only at discrete suture attachment regions, rather than throughout the entire contact surfaces between the device components, to preserve the relative motion of the unconstrained leaflet and skirt regions during deployment and subsequent FSI analysis. The balloon delivery system was meshed with ~ 82,000 unstructured shell elements whereas the geometry was derived from that reported by Bailey et al. (2016). As done for calcific plaques, the linear elastic material properties reported by Bailey et al. (2016) were converted into an equivalent nearly incompressible Neo-Hookean formulation for the balloon delivery system. This conversion led to C1 = 36.5 MPa and D = 1.36E−3 MPa-1.

Once the S3 was assembled with the delivery system, the device was place with 1/3 of the stent frame height below the patient annulus using in the Abaqus/Explicit finite-element solver (v.2021hf7, Dassault Systèmes, FR). Later, the TAVI simulation was performed in different simulation steps and accounting the stress and deformed fields from the previous step. The simulation started with the crimping of the stent frame followed by an elastic recoil prior to balloon inflation. A cylindrical crimping surface was used for radially crimping of the device followed by an elastic recoil (step time of 0.1 s) obtained by removing the contact between S3 and crimper. Balloon expansion was simulated using the fluid-cavity approach in Abaqus/Explicit, prescribing the nominal inflation volume according to the device size, namely 17 mL for the 23 mm S3 and 21 mL for the 26 mm S3. Contact interactions between the device components, native leaflets, calcifications, and aortic root were activated during deployment. During deployment, contact conditions with penalty formulation were enabled between the device components and the patient-specific TAVI model. Specifically, frictionless contact was assumed for interactions among the S3, native leaflets, calcifications, and aortic root, whereas a friction coefficient of 0.1 was assigned only to the contact between the balloon and the S3 device to limit relative sliding during balloon inflation. In the second step, the valve leaflets and sealing skirt were mapped onto the deformed stent configuration using nodal displacement as obtained from the deployment simulation, and the overclosure among components were resolved to obtain the final TAVI simulation scenario for subsequent post-TAVI FSI analysis.

### FSI hemodynamic simulation

**Fig. 1:**
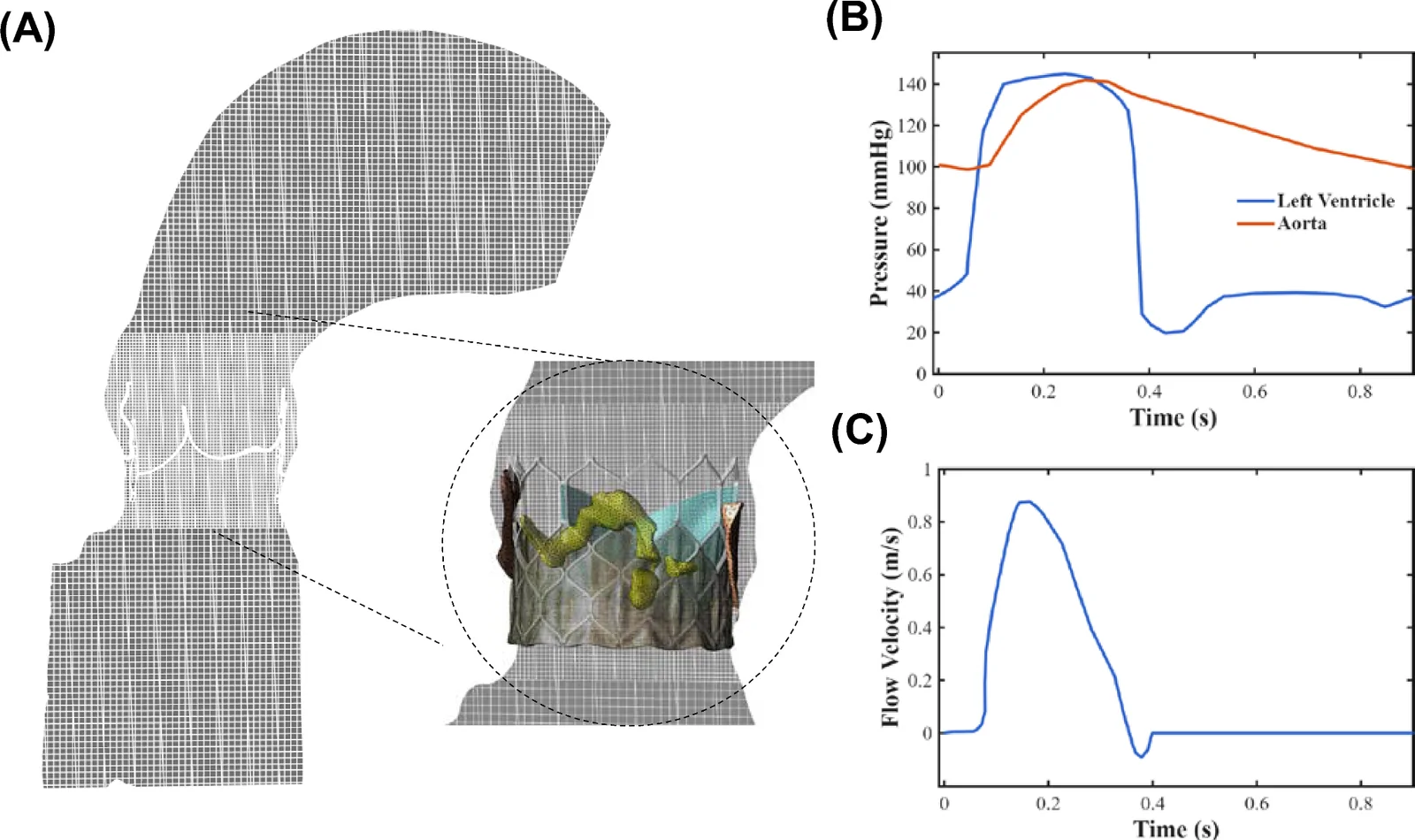
**A** Computational domain for one patient; **B** Patient-specific pressure and flow boundary conditions derived from clinical data

For post-TAVI flow analysis, a fully-coupled two-way FSI simulation was carried out by coupling FlowVision CFD (v3.13.03, Medividia, Belgium) as the fluid domain solver and Abaqus/Explicit as the structural domain solver. The FlowVision software is based on the finite-volume method for the approximations of Navier–Stokes equation for fluid motion. The solver uses a locally adapted mesh with direct representation of curvilinear solid surfaces as fluid neighboring boundaries. This approach ensures conservative data exchange between the solvers and allows for physical contact of the leaflets with full closure. The fluid domain is discretized with a three-dimensional Cartesian mesh with local refinement in correspondence of the complex TAVI geometry as shown in Fig. 1. Mesh sensitivity analysis was aligned with ASME V&V20 and resulted in a uniform grid spacing of 0.6 mm (~ 200,000 cells), yielding a relative error below 1% in the flow quantities. Specifically, mesh sensitivity was assessed for transvalvular flow rate, pressure drop, and peak systolic velocity, but not for wall shear stress-related quantities, which are more sensitive to near-wall resolution and boundary-layer discretization. The FlowVision enabled computation of velocity and pressure in the fluid domain as well as the forces exerted on the S3 valve leaflets with the aortic wall set as rigid. These data are exchanged with the structural solver via the Abaqus co-simulation engine with FlowVsion acting as the master. Time stepping was controlled by a CFL-based (surface CFL = 1 & convective CFL = 20) adaptive scheme with a maximum time step of 1 × 10⁻^4^ s, and numerical convergence was ensured using an algebraic tolerance of 1 × 10^−10^. Artificial compressibility was assigned to moving bodies as a numerical stabilization technique.

Blood was modeled as an incompressible Newtonian fluid (*ρ* = 1,060 kg m⁻^3^, *μ* = 0.004 Pa s) with turbulence accounted using the k–ε FlowVision (KEFV) model (D'Ancona et al. 2013). Though reasonable for large-vessel and high-shear transvalvular flow, the assumption of a Newtonian fluid may affect the estimation of hemodynamic quantities in low-shear and recirculating regions. Furthermore, the k–ε FlowVision turbulence model was adopted in the present FSI simulations because of its numerical robustness within the fully coupled FlowVision–Abaqus workflow. However, other turbulence models may provide improved near-wall flow resolution and should be considered in future studies. Patient-specific flow boundary conditions were imposed switching from velocity to pressure boundary conditions at inlet. Specifically, a general inlet flow velocity waveform was prescribed during systole at inflow with the peak flow scaled according to pre-TAVI echocardiographic transaortic flow measurements (see Fig. 1C). During diastole, the inflow velocity is replaced with a pressure boundary conditions with peak and duration scaled according to patient cuff pressure and heart rate (see Fig. 1B). At the outflow, a pressure waveform profile was adopted for the whole cardiac cycle duration. The inlet and outlet locations were kept consistent across all patient-specific models, as shown in Fig. 1. The pressure and flow waveforms reported in Fig. 1 were used as reference profiles and were scaled for each patient according to the clinical parameters reported in Table 1, including heart rate, systolic and diastolic blood pressure, and echocardiography-derived flow information. We accepted the simulation as dynamically converged if the integral response was within 1% of the previous peak. This was achieved at the beginning of second cardiac cycle if the simulation start point is picked considering the starting configuration of the leaflets. All FSI simulations were computed on Dell R7525 server equipped with two AMD EPYC 75F3 processors using 50 cores for a computation effort of approximately 48 h for two cardiac simulation beats with the last one used for analysis. Additional details of the numerical model verification are reported in our previous two-part study on patient-specific TAVI modeling (Catalano et al. 2025; Scuoppo et al. 2025).

### Hemodynamic analysis and clinical comparison

Numerical estimates of flow velocity, EOA, and peak and mean TPGs, calculated according to ISO-5840 definitions, were compared with post-procedural echocardiographic measurements. This analysis was used to quantify agreement between simulation outputs and routinely available clinical indices, rather than to claim full clinical validation of the model. For qualitative assessment but not for validation purposes, colored maps of blood residence time (BRT) and fluid viscous shear stress at different phase of the cardiac cycle were computed. Regarding BRT, the formulation proposed by Ghirelli and Leckner (2004) was adopted to solve an additional scalar transport equation for the local residence time of the fluid as defined by the time that a fluid particle has spent inside the computational domain since its entry. The scalar was set to zero at the inlet and increased locally with time while being transported by the blood flow. This indicates that low BRT values indicate recently refreshed blood whereas high BRT values identify regions of prolonged residence and potential flow stagnation. The maximum principal stress and time-averaged wall shear stress (TAWSS) were also analyzed. These parameters enable assessments of the region at high risk of thrombus formation or tissue damage of S3 device, which may ultimately lead to early valve failure. For regional analysis, each S3 leaflet was divided along the radial direction from the stent attachment line to the free/coaptation edge. The normalized radial coordinate was defined from 0% at the attachment line to 100% at the leaflet free edge. Three regions were then identified: attachment region, 0–33%; belly region, 33–66%; and free-edge region, 66–100%. The same criterion was applied consistently to the left-coronary, right-coronary, and non-coronary leaflets in all patient-specific models.

For comparison with echocardiographic measurements, the empirical cumulative distribution function (ECDF) curves of the relative errors between prediction and actual clinical measurements were estimated for each hemodynamic quantity. This enables us to perform a one-to-one quantitative comparison showing the proportion of simulation outputs that are less or equal to a given value. Furthermore, the level of agreement was determined using the area metric parameter as defined by the area under the ECDF curves for each quantity of interest. The percentage of area metric was computed as a quantitative parameter of the difference between simulated output and echocardiographic measurements.

## Results

**Fig. 2:**
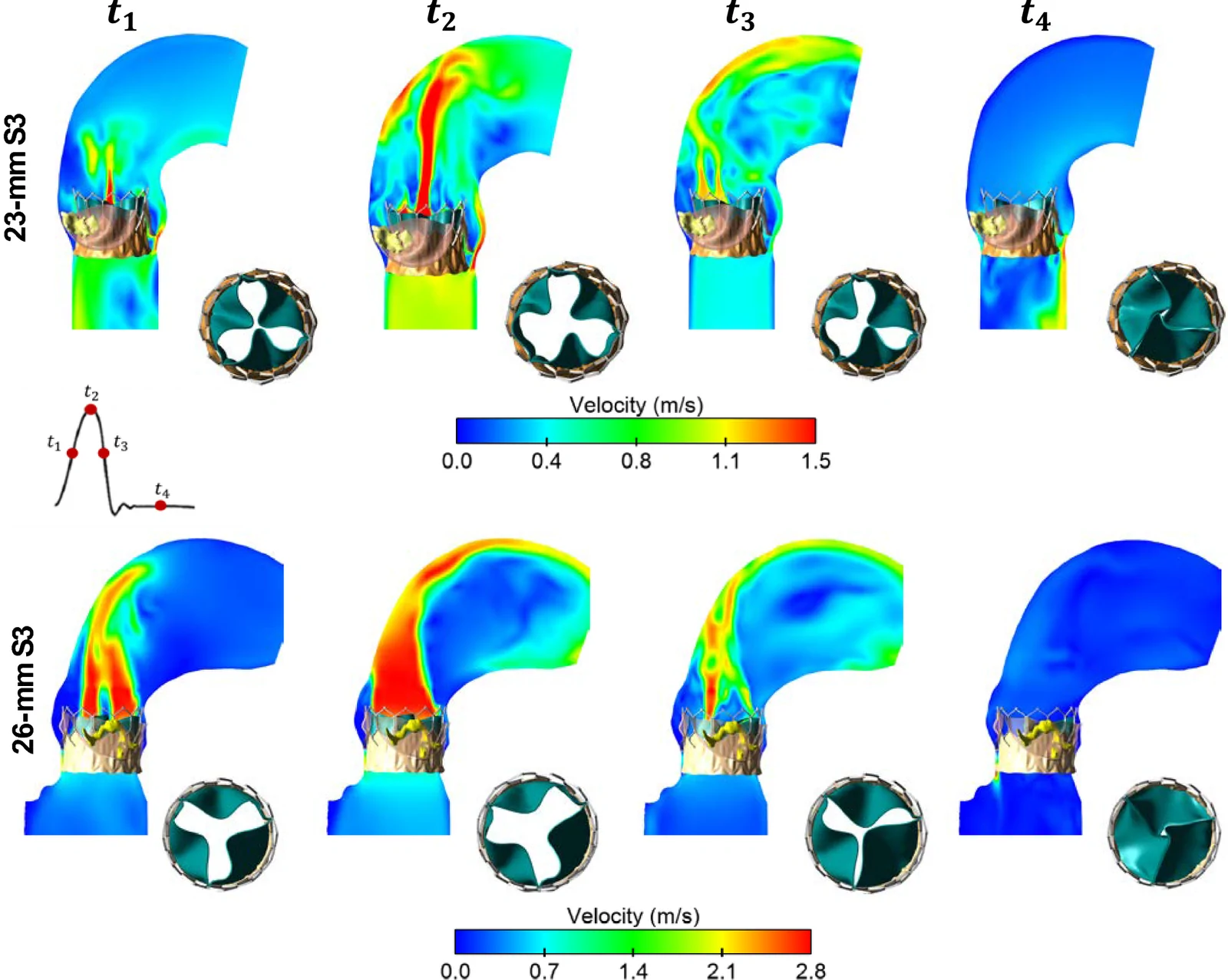
Maps of flow velocity over the cardiac cycle for the patient Case A with the 23 mm S3 and patient Case B with the 26 mm S3. The slice views are shown on a central longitudinal plane passing through the valve axis and are viewed from the non-coronary leaflet side. The time points correspond to: t1, early systolic acceleration; t2, peak systole; t3, systolic deceleration; and t4, diastolic valve closure

**Fig. 3:**
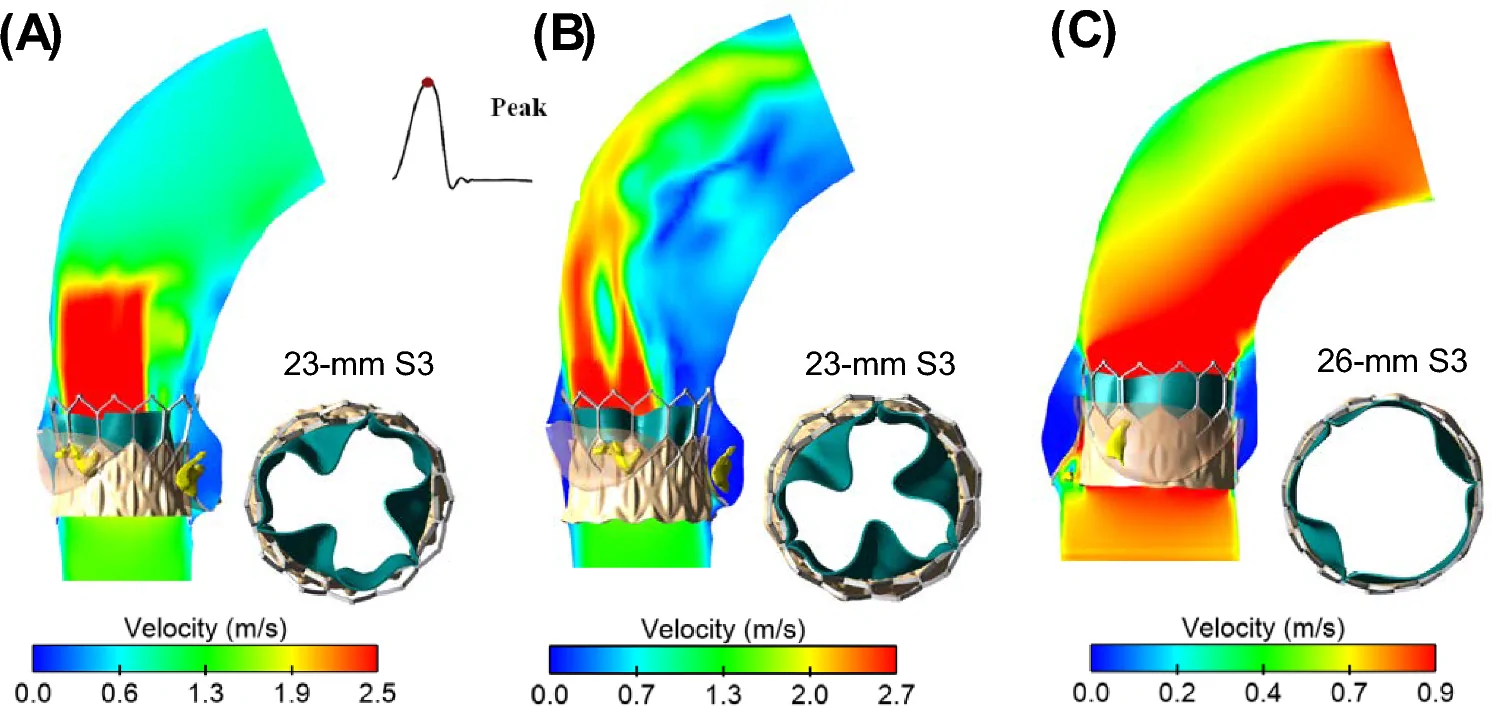
Maps of flow velocity at peak systole for the **A** patient Case C with the 23-mm S3 **B** Case D with the 23 mm S3, and **C** Case E with the 26 mm S3. The slice views are shown on a central longitudinal plane passing through the valve axis and are viewed from the non-coronary leaflet side. The time points correspond to peak systole

**Fig. 4:**
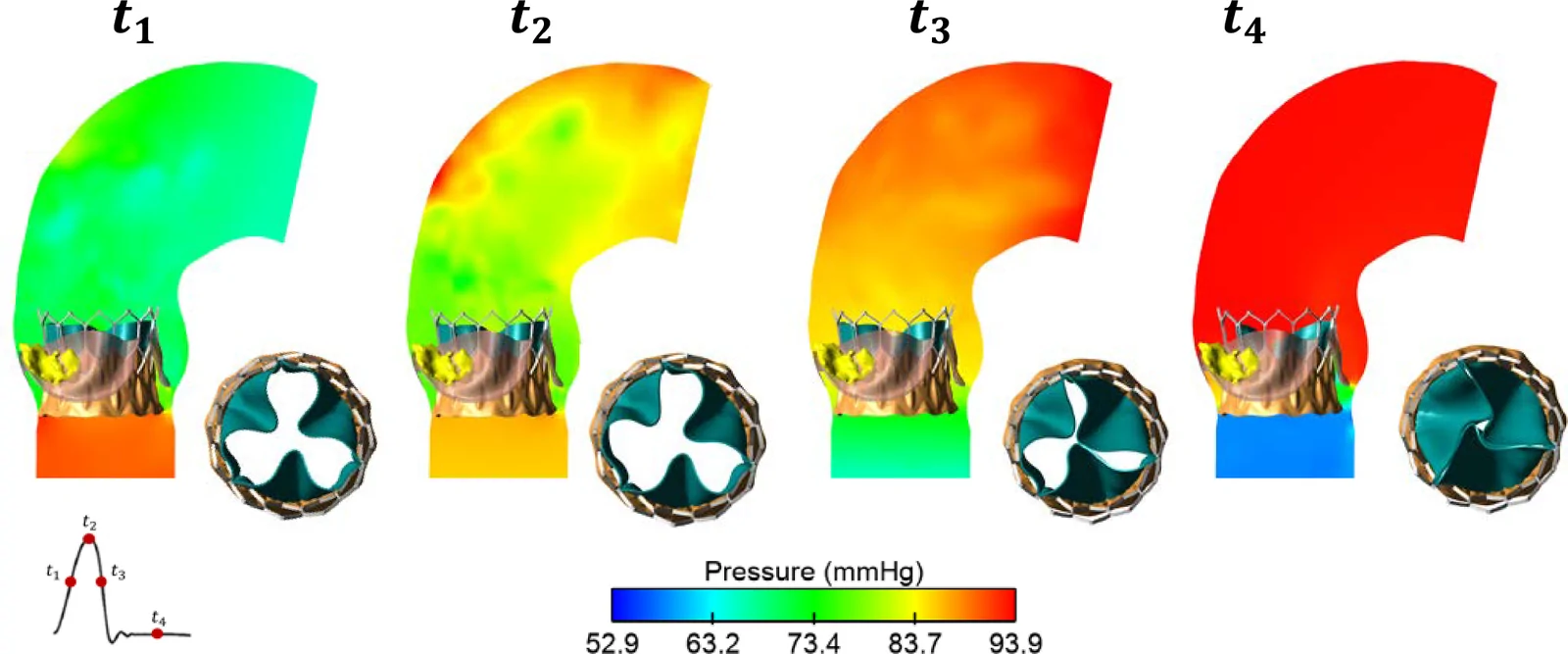
Maps of fluid pressure over the cardiac cycle for the patient Case A with the 23 mm S3. The slice views are shown on a central longitudinal plane passing through the valve axis and are viewed from the non-coronary leaflet side. The time points correspond to: t1, early systolic acceleration; t2, peak systole; t3, systolic deceleration; and t4, diastolic valve closure

Figure 2 illustrates the temporal evolution of flow velocity at different phases of the cardiac cycle for two representative patients with both the 23 mm and 26 mm S3. A hemodynamic environment consistent with expected physiological behavior was observed in all cases. During the acceleration phase, the S3 leaflets progressively opened and generated a well-structured central flow jet during systole. Peak systolic velocity varied among patients, ranging from 1.6 m/s in Case 2 to 3.3 m/s in Case E, with an average value of 2.66 ± 0.59 m/s across the five models. This variability reflected differences in implanted valve size, patient-specific anatomy, and final deployed valve configuration. Valve coaptation was achieved during diastole, with paravalvular leakage qualitatively observed in a few cases. Device valve leaflet closure occurred asymmetrically in all cases because of calcification causing asymmetric expansion of the deployed valve. A similar pattern of flow velocity was shown by other patient-specific TAVI models, as shown in Fig. 3. The distribution of the fluid pressure field throughout the S3 valve leaflets was quantified (see Fig. 4). At different instants of the cardiac cycle, we observed that the aortic and ventricular pressures were homogeneous in space when the valve was fully closed, with the pressure highest in the ventricular outflow tract or the aorta, depending on whether the phase was early systole or late diastole.

**Fig. 5:**
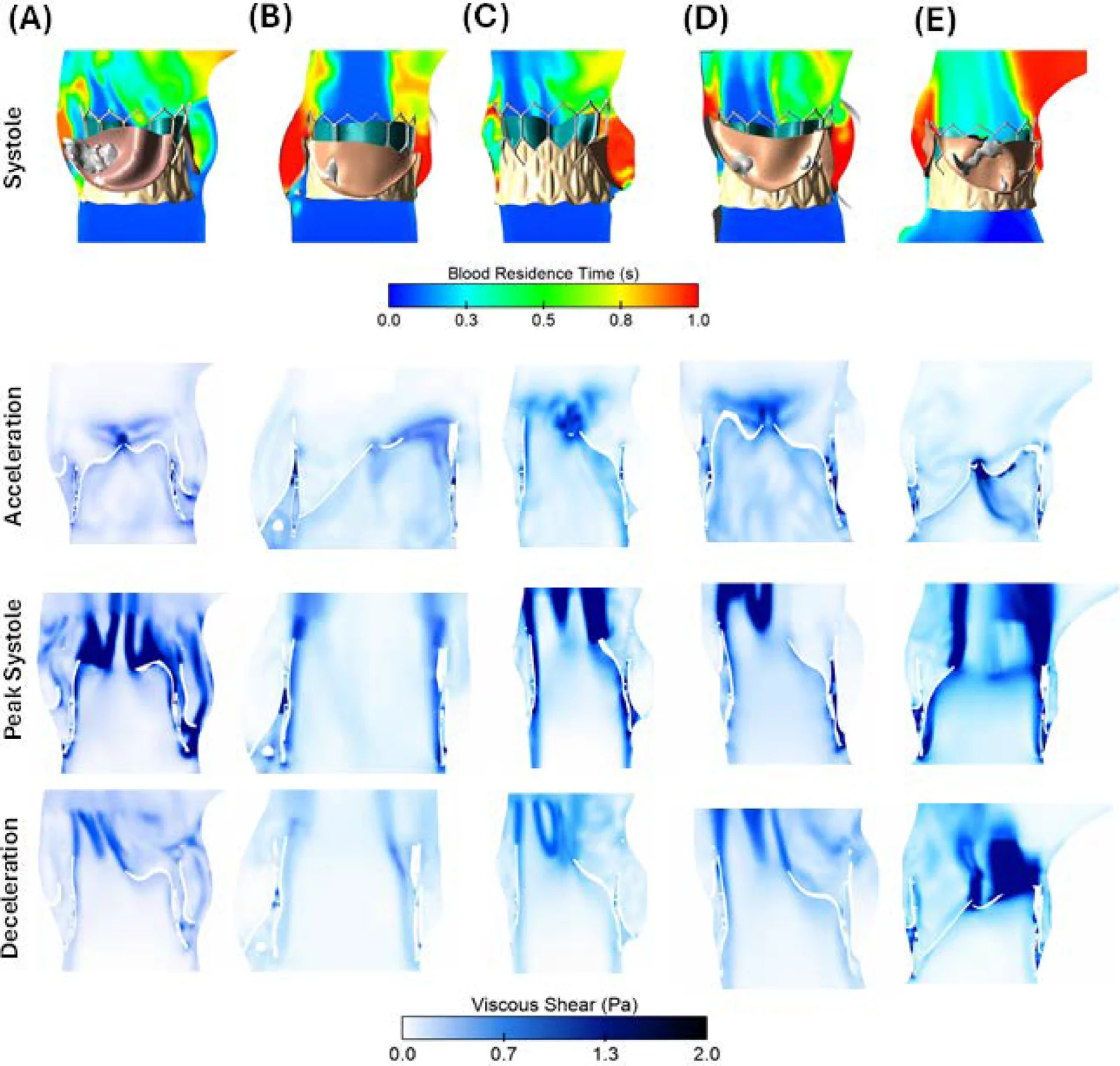
Maps of BRT at peak systole and viscous shear stress at three-time instants of the cardiac cycle for the patient-specific TAVI models with the 23 mm S3 (i.e., Case A,C, and D) and 26 mm S3 (ie, Case B and E). BRT maps are shown at peak systole. Viscous shear stress maps are shown at three representative time points of the cardiac cycle: early systolic acceleration, peak systole, and systolic deceleration. Images within each row correspond to the same cardiac cycle phase across all cases

Figure 5 highlights the distribution of BRT at peak systole and the viscous shear force at three different phases of the cardiac cycle. In all cases, the maximum BRT occurs in the region confined between the displaced stenotic valve leaflets and the S3 device, known as the neo-sinus. This suggests local blood flow stagnation, ultimately leading to an increased thrombogenic risk. In all patient models, fluctuations in viscous shear were observed and the maxima occurred just distal to the free edge of the prosthetic valve leaflets. The highest fluid shear force was observed at peak systole compared to the acceleration and deceleration phases.

**Fig. 6:**
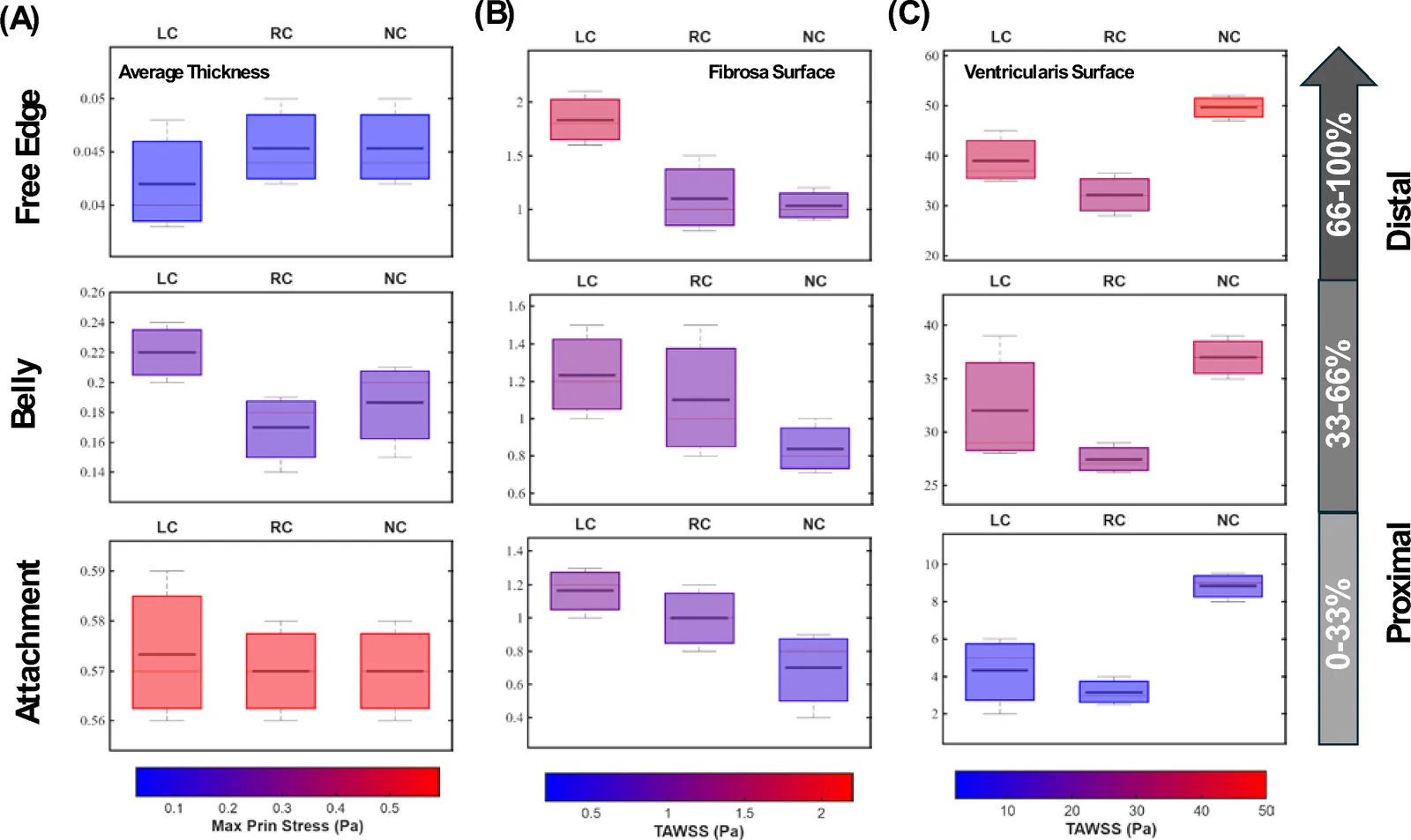
Box plot of maximum principal stress and TAWSS at different height level of each valve leaflet; LC = left-coronary valve leaflets; RC = right-coronary valve leaflet; NC = non-coronary valve leaflets

Further analysis of the stress exerted on the bioprosthesis was performed using box plots (mean ± standard deviation) of the maximum principal stress and TAWSS exerted on each valve leaflet (see Fig. 6). Over the cardiac cycle, the maximum principal stress averaged over the tissue thickness was most pronounced at the attachment of the device valve leaflets to the stent frame (0.578 ± 0.212 MPa for the left-coronary, LC, leaflet, 0.570 ± 0.174 MPa for the right-coronary, RC, leaflet, and 0.569 ± 0.176 MPa for the non-coronary, NC, leaflet). The values then decreased from the belly to the distal free edge, with slight differences among the valve leaflets. In contrast, TAWSS was consistently highest in the free-edge region of the valve leaflets compared with the belly and attachment regions. Furthermore, the ventricularis surface of each valve leaflet exhibited markedly higher TAWSS than the fibrosa surface (i.e., 1.982 ± 0.745 MPa for the LC leaflet on the fibrosa surface versus 39.743 ± 4.529 MPa for the LC leaflet on the ventricularis surface).

**Fig. 7:**
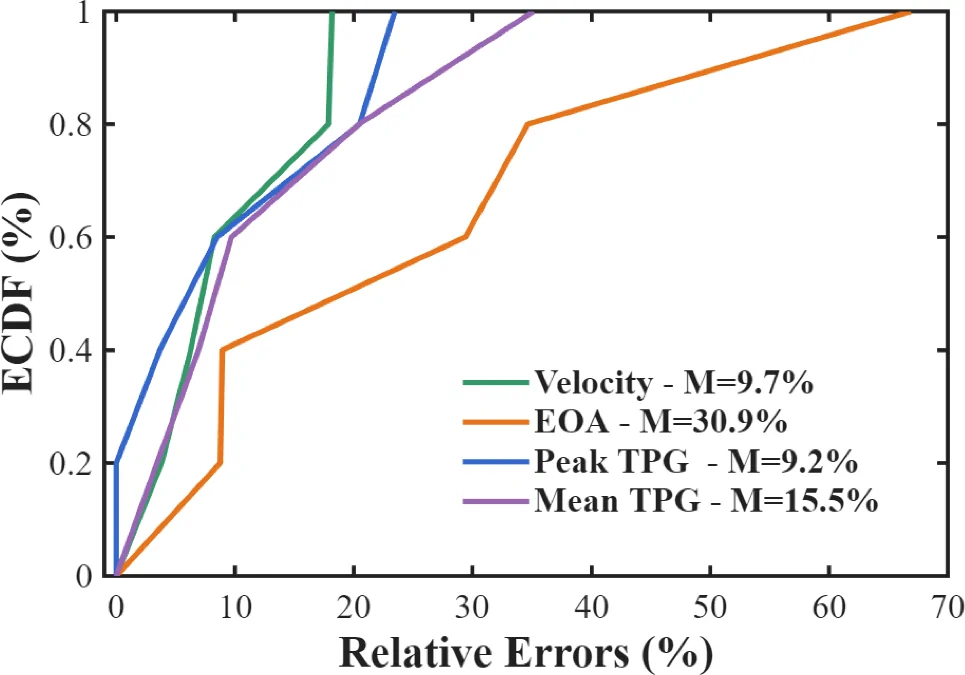
ECDF curves of relative differences between simulation-derived and echocardiographic hemodynamic indices, with corresponding area metrics

The ECDF curves of the relative differences between in silico and echocardiographic quantities of interest were computed to describe the level of agreement between simulation outputs and clinical measurements and thus quantify the model predictive capabilities (see Fig. 7). The transaortic flow velocity from the S3 at systole was 2.66 ± 0.59 m/s and closely matched the echocardiographic measurement of 2.68 ± 0.42 m/s. The predicted EOA of 1.88 ± 0.54 cm^2^ contrasted with the clinical value of 1.42 ± 0.21 cm^2^, whereas the predicted TPG was 22 ± 10.7 mmHg compared with the in vivo value of 22.4 ± 10.96 mmHg. The area metric calculations yielded values of 9.7% for velocity, 30.9% for EOA, 9.2% for peak TPG, and 15.5% for mean TPG.

## Discussion

As TAVI has been increasingly adopted over the past decades, in silico FSI approaches have been proposed to capture the interaction between blood flow and the prosthesis, thereby enabling an in-depth evaluation of TAVI outcomes across patient population (Vasileios et al. 2024). However, the dynamic nature of valve kinematics and the need for quantifying model predictive performance pose challenges for translating basic knowledge into a valuable medical device software for TAVI planning. Furthermore, TAVI procedures are expanding to younger patients, yet the durability of transcatheter heart valves may fail to meet lifetime requirements. Structural valve degeneration due to subclinical leaflet thrombosis can occur in the absence of remarkable changes in transvalvular gradients (Blackman et al. 2019). These considerations prompted us to develop a patient-specific FSI computational model of post-TAVI hemodynamics and to perform a preliminary comparison of selected simulation-derived indices with post-procedural echocardiographic measurements. Five patient models treated with either the 23 mm or 26 mm S3 device were developed in accordance with the verification activities suggested by the ASME V&V 40 standard. Patient-specific anatomies were reconstructed from pre-procedural CT scans. All procedural steps of TAVI were realistically reproduced, including pre-dilatation of the stenotic native valve, device crimping, and in situ device expansion, followed by a fully coupled two-way FSI analysis based on personalized boundary conditions to account for physiological variability. The FSI model was capable of estimating structural and hemodynamic parameters throughout the post-TAVI cardiac cycle, with selected hemodynamic outputs compared against post-procedural echocardiographic indices to evaluate their consistency with routinely acquired clinical measurements.

The assessment of ECDFs of relative differences provides a means to visualize the distribution of discrepancies between simulation-derived and echocardiographic measurements across the five cases. For the peak TPG across the S3, the ECDF approached high levels (close to 1) at modest values of relative error (nearly 20% with four data points). This indicates that the majority of simulation data points (i.e., patient models) had relative errors within 20% of the in vivo data. In contrast, for the EOA, many ECDF data points exhibited relatively high errors. This suggests that the FSI predictions often deviated from the actual clinical measurements. These findings are reflected in the area metric, which was used as a single parameter to quantify the degree to which the ECDF distributions fit, rather than focusing on differences from a single data point. We found that the predictions of peak systolic flow velocity and TPG remained below 10% error with respect to echocardiographic measurements, whereas the EOA showed substantial discrepancies.

There are two possible explanations for the discrepancy in the predicted EOA. First, patient-specific anatomical differences may influence stent deployment leading to asymmetric valve expansion and thus orifice area lower than the nominal one. Second, the reliability of echocardiography-based EOA measurements can be affected by acoustic window limitations, poor image quality in some patients, and misalignment of the Doppler beam with the flow direction. In contrast, the FSI simulations enabled direct estimation of EOA. Indeed, Ghosh et al. (2020b) specifically highlighted that while clinical measurements are limited by 1D assumptions, FSI simulations are based on 3D physics that capture the complete fluid domain more faithfully. Oks et al. (2022) noted that the EOA calculated via Gorlin’s formula is essentially derived from Bernoulli’s law by neglecting the viscous term, which makes the clinical metric inherently less accurate than a geometric or full-physics simulation. Here, we found that FSI-based estimates of flow quantities are consistent with previous studies (Vogl et al. 2022; Hatoum et al. 2022). Vogl and colleagues (2022) assessed S3 flow performance in a pulse duplicator via particle image velocimetry. The resulting EOA was 1.81 ± 0.05 cm^2^, compared with our mean value of 1.88 ± 0.59 cm^2^, and the mean TPG was 10.56 ± 0.62 mmHg compared with our estimate of 15.28 ± 8.05 mmHg. Our peak velocity reached 2.66 ± 0.59 m/s, which is comparable to the 2.8 m/s reported by Hatoum et al. (2022), who also reported an EOA of 2.06 ± 0.03 cm^2^ for the S3.

The proposed fully-coupled two-way coupled FSI framework enables the quantification of hemodynamic and structural descriptors that are not directly measurable in vivo. The adopted boundary-conforming approach ensured accurate resolution of near-wall gradients and transient flow phenomena, allowing reliable characterization of jet impingement regions and recirculation patterns. In agreement with a previous study (Mao et al. 2016), elevated WSS values were observed at the jet impingement zone. BRT distributions exhibited inter-patient variability, with higher values predominantly localized within the neo-sinus and along the aortic curvature. From a biomechanical standpoint, the combined evaluation of structural loads and hemodynamic descriptors provides a mechanistic framework to investigate valve durability and thrombogenic risk. In contrast to FE-only models, which may underestimate cyclic behavior of the bioprosthesis, the present FSI formulation captures the water hammer effect at valve closure. This is considered a transient phenomenon associated with increases in peak stresses of up to 28% as suggested by Mao et al. (2016). However, the water hammer effect at valve closure in our study is likely overestimated due the absence of the coronary arteries and the assumption of a rigid aorta. Regions exposed to elevated TAWSS were identified as potential sites of structural valve deterioration, consistent with correlations reported in long-term follow-up studies by Fumagalli et al. (2023). They identified a direct correlation between TAWSS critical areas (> 0.5 Pa) on the proximal aortic wall and actual structural valve deterioration observed in patients during 5–10 year follow-ups. The integration of BRT also enabled the identification of stagnation-prone regions within the neo-sinus, supporting the characterization of patient-specific thrombogenic footprints. Here, we incorporated several aspects that strengthen physiological realism. First, patient-specific boundary conditions were calibrated using individual clinical measurements to avoid the use of generalized inflow profiles. Secondly, comparison using echocardiographic data was implemented using a probabilistic assessment in accordance with principles suggested by the ASME V&V 40 framework. In a similar study, Grossi et al. (2024) established credibility through a dual-validation approach, first calibrating Nitinol material properties via experimental crimping tests and then quantitatively comparing simulated stent configurations against post-operative CT scans. They achieved mean differences of only 1.79% for orifice area between in silico and in vitro. Luraghi et al. (2019) performed a comprehensive clinical validation of their FSI methodology by demonstrating a close qualitative match between simulated device positioning and post-operative imaging, while also quantitatively matching numerical velocity fields with patient-specific Doppler traces.

The primary limitation of this study was the small number of FSI simulations. The assumption of a rigid aortic wall may have affected the hemodynamic findings, though aortic wall displacement was found to be minimal in another FSI study done by our group using smoothed-particle hydrodynamics (Catalano et al. 2025). Paravalvular leakage was evaluated qualitatively from the FSI flow fields. Quantitative leakage analysis was not performed because clinical echocardiographic grading was qualitative, limiting the possibility of direct comparison with simulation-derived leakage volumes. Another limitation is that the virtual deployment accuracy was not directly assessed against post-TAVI CT imaging in the same five patients included in the present FSI analysis. As a result, possible errors in the predicted deployed valve configuration may have influenced the subsequent hemodynamic comparison. Unlike studies that include direct post-operative CT-based device-configuration comparison or catheter-based pressure measurements, the present study relied on routinely available echocardiographic metrics. Therefore, the comparison reported here should be regarded as preliminary clinical benchmarking rather than a real clinical validation. Future work should include larger cohorts, direct post-TAVI imaging of deployed device configuration, catheter-based pressure gradients where available, and direct comparison between simulated velocity fields. Despite these limitations, the present framework establishes a scalable FSI-related method to deploy simulations in large cohort of TAVI patients.

## Conclusion

This study demonstrates the feasibility of a fully coupled, patient-specific FSI framework for post-TAVI analysis of S3 Ultra hemodynamics and valve leaflet mechanics. The framework reproduced patient-specific flow patterns and enabled quantification of structural and hemodynamic descriptors not directly measurable in vivo. A preliminary comparison with post-procedural echocardiographic measurements showed good agreement for peak velocity and peak TPG, whereas EOA showed larger discrepancies. These findings support the potential value of the approach for mechanistic post-TAVI assessment, while highlighting the need for larger-cohort studies and more direct clinical datasets before claims of clinical validation or patient-specific predictive use can be made.

## Data Availability

No datasets were generated or analysed during the current study.
